# Occupational Pesticide Exposure and Risk of Non-melanoma Skin Cancer Among U.S. Adults: A Systematic Review

**DOI:** 10.7759/cureus.116415

**Published:** 2026-09-17

**Authors:** Daad G Alrowaili, Wendy Miranda, Uchechi A Nwaneri, Tariro T Nyamandi, Kofi Effa-Ababio, Sarah Waseem

**Affiliations:** 1 Genetics and Human Genetics, Howard University, Washington, DC, USA; 2 General Surgery, University of Ghana Medical Center, Accra, GHA; 3 Internal Medicine, Howard University Hospital, Washington, DC, USA; 4 Internal Medicine, University of Zimbabwe, Harare, ZWE; 5 Investigational Cancer Therapeutics, The University of Texas MD Anderson Cancer Center, Houston, USA; 6 General Medicine, Dow University of Health Sciences, Karachi, PAK; 7 Rehabilitation Science, McMaster University, Hamilton, CAN

**Keywords:** agricultural workers, basal cell carcinoma, non-melanoma skin cancer, occupational pesticide exposure, squamous cell carcinoma, systematic review

## Abstract

Non-melanoma skin cancer (NMSC), including basal cell carcinoma (BCC) and squamous cell carcinoma (SCC), is the most common cancer diagnosed in the United States. Although ultraviolet (UV) radiation is the major risk factor, occupational exposure to pesticides has been proposed as an additional contributor among agricultural and other outdoor workers. This systematic review evaluated the available evidence on the association between occupational pesticide exposure and the risk of NMSC among U.S. adults. Following PRISMA 2020 guidelines, PubMed, the Excerpta Medica database (Embase), Web of Science, and the Cochrane Library were searched for eligible observational studies published between 2015 and March 2026. Study quality was assessed using the Newcastle-Ottawa Scale (NOS). The search yielded 490 records, and 26 studies met the inclusion criteria. Across the included studies, workers with prolonged pesticide exposure, frequent dermal contact, longer employment in agricultural settings, and inconsistent use of personal protective equipment generally had a higher risk of NMSC. Several studies also described biological mechanisms, including oxidative stress, chronic inflammation, and DNA damage, that may explain this relationship. However, differences in exposure assessment and the influence of concurrent ultraviolet exposure limited direct comparisons between studies. Current evidence supports an association between long-term occupational pesticide exposure and increased NMSC risk, but additional prospective studies using more accurate exposure measurements are needed to strengthen the evidence.

## Introduction and background

Non-melanoma skin cancer (NMSC), predominantly basal cell carcinoma (BCC) and squamous cell carcinoma (SCC), remains an important public health concern in the United States. In parallel with the substantial burden of skin cancer, occupational exposure to pesticides has received increasing attention because of the potential carcinogenic effects associated with long-term exposure. Evidence from the Agricultural Health Study (AHS) has examined cancer incidence among pesticide applicators in relation to several commonly used pesticides, including acetochlor [1], diazinon [2], and metolachlor [3]. Although these studies contribute to understanding the carcinogenic potential of occupational pesticide exposure, evidence concerning NMSC specifically remains limited.

Ultraviolet (UV) radiation is a well-established environmental determinant of skin cancer and is particularly relevant when considering workers who spend substantial time outdoors [4]. U.S. epidemiological studies have also demonstrated the considerable occurrence of BCC and variation in its distribution and risk factors [5,6]. This background is important in occupational studies because pesticide exposure rarely occurs independently of other cancer-related factors. Evidence from the AHS has examined occupational pesticide exposure in relation to cancer incidence [7], while population-based data have documented trends in the incidence of both BCC and cutaneous SCC [8]. Further pesticide-specific analyses have evaluated glyphosate exposure and cancer incidence among licensed pesticide applicators [9]. At the same time, individual susceptibility remains important, as demographic and other risk factors have been associated with BCC occurrence [10].

The occupational setting adds further complexity to this relationship. Agricultural and other outdoor workers may experience substantial UV exposure alongside pesticide exposure, making it difficult to distinguish the contribution of individual occupational hazards. Studies of agricultural and construction workers have identified variation in skin cancer prevention and sun-protection practices [11]. Long-term follow-up of the AHS has provided additional information on cancer incidence among pesticide applicators [12], including analyses of individual agents such as dicamba [13]. Studies of agricultural workers have also identified gaps in skin cancer prevention and screening practices [14]. These findings are relevant because differences in protective behavior, together with age, sex, race, and geographic location, may influence the occurrence and distribution of BCC and SCC [15]. The identification of risk factors among individuals with frequent BCC further illustrates the importance of considering individual susceptibility when interpreting occupational associations [16].

Exposure characterization is another important consideration. Prospective occupational studies increasingly incorporate information on pesticide type, duration and frequency of use, and cumulative exposure. Investigations of pyrethroid use within the AHS provide additional evidence concerning the long-term health effects associated with occupational insecticide exposure [17]. Biomonitoring approaches have also strengthened the assessment of biological responses to pesticide exposure. In the AHS, glyphosate exposure has been associated with urinary biomarkers of oxidative stress [18], providing mechanistic evidence relevant to pesticide-associated carcinogenesis, although such findings do not establish a direct association with NMSC.

Taken together, the available literature presents two related but not fully integrated bodies of evidence: studies examining occupational pesticide exposure and broader cancer outcomes, and studies characterizing NMSC incidence, risk factors, and occupational skin cancer prevention. Relatively few studies have directly examined occupational pesticide exposure in relation to BCC or SCC among U.S. adults. This distinction represents the principal knowledge gap addressed by the present review. Accordingly, this systematic review aimed to synthesize the available direct and supporting observational evidence relevant to the association between occupational pesticide exposure and NMSC risk among U.S. adults, while considering occupational, environmental, and individual factors that may influence this relationship.

## Review

Materials and methods

Eligibility Criteria and Search Strategies

This systematic review was conducted as per the PRISMA 2020 guidelines with an aim of ensuring transparency, reproducibility, and methodological rigor [19]. The study carefully selected articles that were peer-reviewed and published between 2015 and March 2026. The main databases used in the search for the articles comprised the Excerpta Medica database (Embase), Web of Science, PubMed, and Cochrane Library. The search strategy was conducted using the Population, Intervention, Control, and Outcomes (PICO) framework components [20]. Some of the keywords used in the search for these articles included the following: occupational pesticide exposure, non-melanoma skin cancer, basal cell carcinoma (BCC), squamous cell carcinoma (SCC), agricultural workers, pesticides, occupational exposure, and the United States (Table 1).

**Table 1:** Overview of the search strategy Embase: Excerpta Medica database

Category	Details
Databases Searched	Embase, Web of Science, PubMed and Cochrane Library
Time Frame	Studies published between 2015 and 2026 were included
Language	English only
Search Strategy Structure	The search strategy was structured using a combination of four key components; #1, #2, #3 and #4
#1 (Population)	Adults OR agricultural workers OR farmworkers OR pesticide applicators OR occupationally exposed workers OR forestry workers OR landscapers OR pest control workers OR
#2 (Exposure/Risk Factors/Intervention)	Occupational pesticide exposure OR pesticides OR herbicides OR insecticides OR agricultural chemicals
#3 (Study Context)	United States OR U.S. adults OR U.S. workers
#4 (Outcome)	Non-melanoma skin cancer OR keratinocyte carcinoma OR basal cell carcinoma (BCC) OR squamous cell carcinoma (SCC) OR skin neoplasms OR skin cancer incidence

Inclusion Criteria

Studies published between January 2015 and March 2026 were considered eligible. Eligible studies were observational investigations, including cohort, case-control, and cross-sectional studies, conducted among adults in the United States. Studies were included if they contributed directly or indirectly to evaluating the relationship between occupational pesticide exposure and skin cancer risk.

Studies contributing direct evidence examined occupational pesticide or agrochemical exposure in relation to skin cancer or NMSC outcomes. Studies contributing supporting evidence evaluated pesticide-specific carcinogenicity, NMSC epidemiology, UV radiation exposure, occupational skin-protection practices, demographic or occupational risk factors for NMSC, or biological mechanisms relevant to pesticide-associated carcinogenesis. Supporting studies were included to assist in the interpretation of confounding factors, effect modifiers, biological plausibility, and the broader occupational context of the pesticide-skin cancer relationship.

Eligible studies were restricted to observational designs suitable for quality assessment using the Newcastle-Ottawa Scale (NOS) or an adapted NOS for cross-sectional studies [21, 22]. Given differences in study design, reported measures of association could include odds ratios (ORs), risk ratios (RRs), incidence rate ratios, hazard ratios (HRs), prevalence ratios, or prevalence estimates. Where available, corresponding 95% confidence intervals were extracted.

Studies published outside the predefined period, conducted exclusively outside the United States, involving pediatric populations, or consisting of reviews, editorials, case reports, conference abstracts, or other non-observational publications were excluded.

Study Selection Process

All studies obtained from the database search were entered into a reference manager program, and duplicates were removed. Two separate reviewers independently compared the titles and abstracts of all studies that were returned from our database search to determine each study's eligibility based on the previously defined eligibility criteria. Full-text articles of studies that appeared to be relevant or for which eligibility was unclear were evaluated next by both reviewers. When there was disagreement between the reviewers regarding the inclusion of a study, it was resolved through discussion and, if needed, with a third reviewer. The study selection was documented using the PRISMA flow diagram.

Quality Assessment

Methodological quality for included studies was assessed using the NOS for observational studies and other appropriate tools for randomized studies. Studies were assigned a risk of bias score based on selection, comparability, and outcome assessment [21, 22].

Data Synthesis

A narrative synthesis was performed because substantial clinical and methodological heterogeneity across the included studies precluded meaningful quantitative pooling. Studies differed in design, occupational populations, pesticide types, exposure assessment methods, exposure duration and frequency, NMSC outcomes, and measures of association. Effect estimates were therefore summarized according to the measures reported by individual studies, including ORs, RRs, incidence rate ratios, and HRs, together with 95% confidence intervals where available. Differences in pesticide exposure characteristics, occupational category, UV radiation exposure, personal protective equipment (PPE) use, and adjustment for potential confounders were considered when interpreting findings. Because no meta-analysis was performed, statistical heterogeneity measures such as I² and Cochran’s Q, pooled effect estimates, and formal publication-bias analyses were not calculated.

Data Extraction

Data extracted from each study included study design, sample size, participant characteristics, occupational category, pesticide type, exposure assessment method, duration and frequency of exposure, NMSC outcome, effect estimates (OR, RR, incidence rate ratio, or HR), corresponding 95% confidence intervals where available, and covariates included in adjusted analyses. Information on UV radiation exposure and PPE use was also extracted when reported.

Results

The database search yielded 490 records. After removal of 90 duplicates, 400 unique records remained for title and abstract screening. Of these, 280 records were excluded because they did not meet the predefined eligibility criteria, leaving 120 articles for full-text assessment. Following full-text review, 94 articles were excluded because they did not meet the predefined criteria regarding study design, population, geographic setting, exposure or outcome relevance, or availability of sufficient data for the evidence synthesis. A total of 26 observational studies were therefore included in the qualitative evidence synthesis (Figure 1).

**Figure 1:**
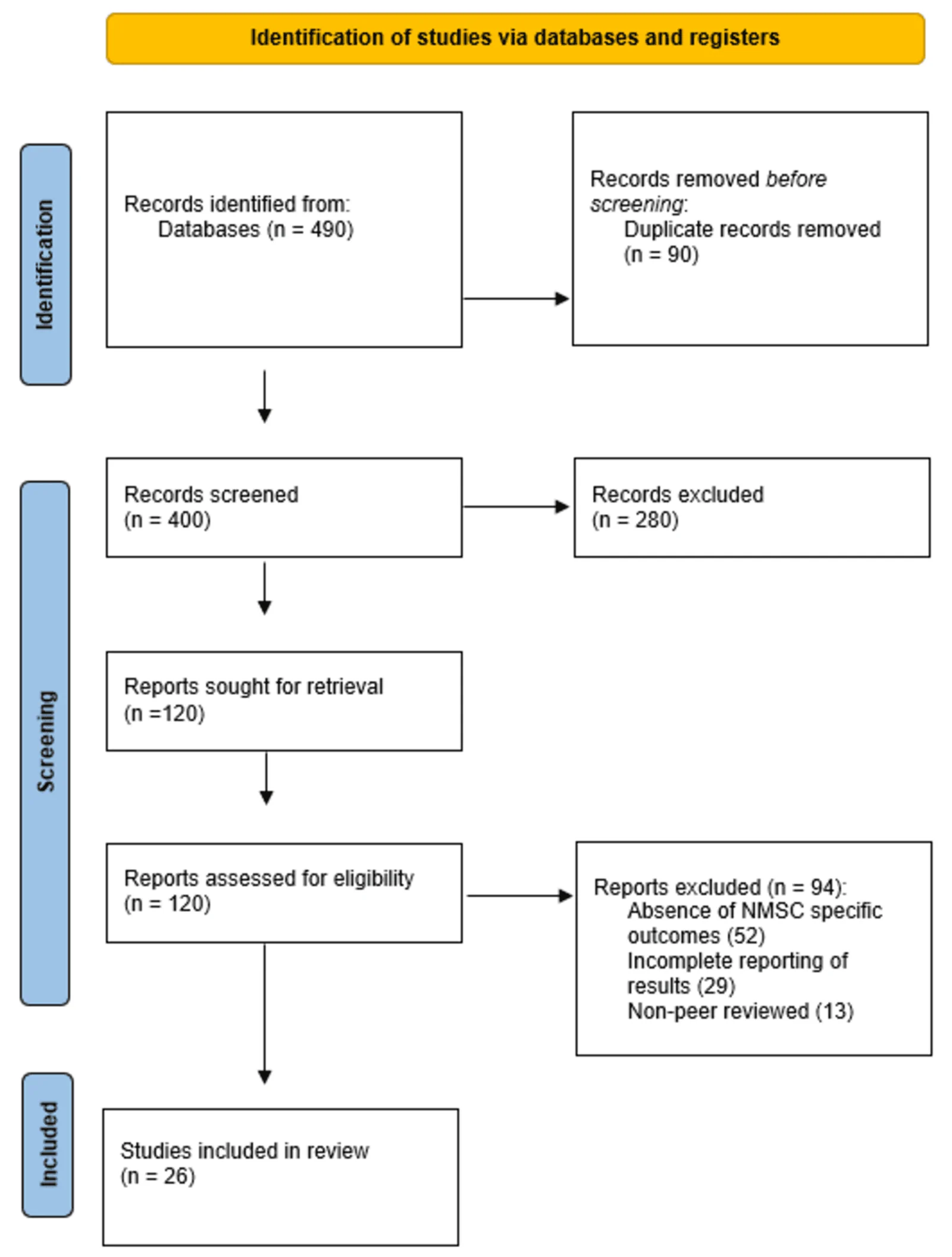
PRISMA flow diagram showcasing the study selection and inclusion process This review was conducted in accordance with the PRISMA 2020 guidelines [19]. This work is licensed under CC BY 4.0. NMSC: non-melanoma skin cancer

The 26 included studies were categorized according to their role in addressing the review question. Direct evidence studies evaluated occupational pesticide or agrochemical exposure in relation to skin cancer outcomes, whereas supporting evidence studies addressed factors relevant to interpretation of this relationship, including pesticide-specific carcinogenicity, NMSC incidence and epidemiology, UV radiation exposure, occupational skin-protection practices, demographic and occupational risk factors, and biological mechanisms relevant to pesticide-associated carcinogenesis. Supporting studies were used to contextualize potential confounding, effect modification, and biological plausibility and were not interpreted as direct estimates of the association between occupational pesticide exposure and NMSC. 

Table 2 presents the summary characteristics of the 26 included studies in this study.

**Table 2:** Summary characteristics of the included studies Direct evidence studies evaluated occupational pesticide or agrochemical exposure in relation to skin cancer outcomes. Supporting evidence studies addressed pesticide-specific carcinogenicity, NMSC epidemiology, UV exposure, occupational skin-protection practices, individual risk factors, economic burden, or biological mechanisms relevant to interpretation of the pesticide–skin cancer relationship. Supporting studies were used to contextualize potential confounding, effect modification, and biological plausibility and were not interpreted as direct estimates of the association between occupational pesticide exposure and NMSC. AHS: Agricultural Health Study; BCC: basal cell carcinoma; CI: confidence interval; cSCC: cutaneous squamous cell carcinoma; MEPS: Medical Expenditure Panel Survey; NMSC: non-melanoma skin cancer; OR: odds ratio; CI: confidence interval; SCC: squamous cell carcinoma; U.S.: United States; UV: ultraviolet.

Study (First Author, Year)	Study Design	Population	Exposure or Factor Evaluated	Outcome	Key Finding
Lerro et al. (2015) [1]	Prospective cohort	Licensed pesticide applicators, AHS	Acetochlor use	Cancer incidence	Evaluated cancer incidence in relation to occupational acetochlor use; contributes pesticide-specific carcinogenicity evidence rather than a direct NMSC estimate.
Jones et al. (2015) [2]	Prospective cohort	Male pesticide applicators, Iowa and North Carolina, AHS	Diazinon use	Solid-tumor incidence	Updated evaluation of diazinon exposure and solid tumors among pesticide applicators; relevant to long-term pesticide-associated cancer risk.
Silver et al. (2015) [3]	Prospective cohort	Licensed pesticide applicators, AHS	Metolachlor use	Cancer incidence	Examined cancer incidence according to metolachlor exposure; provides pesticide-specific cancer evidence rather than direct evidence for NMSC.
Ransohoff et al. (2016) [4]	Prospective cohort	U.S. postmenopausal women, Women's Health Initiative	Residential UV exposure	Skin cancer risk	Demonstrated the importance of UV exposure in skin cancer risk, supporting consideration of UV as a potential confounder in occupational populations.
Goldenberg et al. (2016) [5]	Retrospective cohort	56,987 commercially insured U.S. adults with BCC claims	Demographic/clinical characteristics	BCC incidence and prevalence	Characterized the incidence and prevalence of BCC in a large U.S. population, providing epidemiological context for the review.
Barton et al. (2016) [6]	Population-based case-control	Adults in New Hampshire	Individual and demographic risk factors	Early-onset BCC	Identified characteristics associated with early-onset BCC, emphasizing individual susceptibility when interpreting occupational risk.
Bonner et al. (2017) [7]	Prospective cohort	AHS pesticide applicators	Occupational pesticide exposure	Lung cancer incidence	Evaluated pesticide exposure in relation to lung cancer, contributing evidence on pesticide-associated carcinogenicity but not directly addressing NMSC.
Muzic et al. (2017) [8]	Population-based retrospective cohort	Olmsted County, Minnesota	Demographic/time trends	BCC and SCC incidence	Documented population-level patterns and trends in BCC and SCC incidence, providing NMSC epidemiological context.
Andreotti et al. (2018) [9]	Prospective cohort	54,251 licensed pesticide applicators, AHS	Glyphosate use	Cancer incidence	Evaluated glyphosate exposure and cancer incidence during extended follow-up; not interpreted as direct evidence of pesticide-associated NMSC.
Nemer et al. (2018) [10]	Case-control	U.S. men younger than 40 years	Individual risk factors	BCC	Examined risk factors for BCC in younger men, contributing evidence concerning individual susceptibility to BCC.
Ragan et al. (2019) [11]	Cross-sectional	2,298 U.S. agricultural and construction workers	Occupational UV exposure and protective behavior	Skin cancer prevention	Identified variation in sun-protective practices among outdoor workers, highlighting UV exposure and prevention behavior as relevant occupational factors.
Lerro et al. (2019) [12]	Prospective cohort	AHS pesticide applicators and spouses	Long-term occupational pesticide exposure	Cancer incidence	Extended AHS follow-up characterized long-term cancer incidence in an occupationally pesticide-exposed population.
Lerro et al. (2020) [13]	Prospective cohort	49,922 pesticide applicators, AHS	Dicamba use	Cancer incidence	Examined cancer incidence according to occupational dicamba exposure, adding pesticide-specific evidence to the broader carcinogenicity literature.
Bormann et al. (2020) [14]	Cross-sectional survey	Midwestern U.S. agricultural workers	Occupational/behavioral factors	Skin cancer prevention and screening	Identified skin cancer prevention and screening behaviors among agricultural workers, relevant to potential confounding by UV exposure and protective practices.
Lukowiak et al. (2020) [15]	Retrospective observational study	U.S. patients with keratinocyte carcinoma	Age, sex, race, and geographic region	BCC-to-cSCC ratio	Demonstrated demographic and geographic variation in the distribution of BCC and cSCC.
Chiang et al. (2021) [16]	Observational study	U.S. patients with BCC	Demographic and clinical risk factors	High-frequency BCC	Characterized factors associated with frequent BCC occurrence, providing additional evidence on individual susceptibility.
Shrestha et al. (2022) [17]	Prospective cohort	AHS participants	Permethrin and other pyrethroid use	Long-term mortality outcomes	Evaluated long-term outcomes associated with occupational pyrethroid exposure; relevant to pesticide safety but not a direct estimate of NMSC risk.
Chang et al. (2023) [18]	Biomonitoring observational study	AHS participants	Glyphosate exposure	Urinary oxidative-stress biomarkers	Glyphosate exposure was associated with oxidative-stress biomarkers, providing mechanistic evidence relevant to pesticide-associated carcinogenesis.
Kao et al. (2023) [23]	Cross-sectional/healthcare expenditure analysis	U.S. adults, MEPS	Skin cancer diagnosis/treatment	Economic burden of skin cancer	Demonstrated the healthcare and economic burden associated with skin cancer treatment in the United States.
Remigio et al. (2024) [24]	Prospective cohort	53,562 pesticide applicators, AHS	Atrazine use	Cancer incidence	Updated atrazine–cancer associations in pesticide applicators, contributing pesticide-specific evidence relevant to occupational carcinogenicity.
Tucker et al. (2025) [25]	Cross-sectional observational study	7,943 farmers and ranchers in seven U.S. states	Dermal pesticide and fertilizer exposure	Physician-diagnosed skin cancer	Dermal exposure to pesticides and fertilizers was associated with higher odds of skin cancer (adjusted OR 1.30, 95% CI 1.08–1.56).
Vanaria et al. (2025) [26]	Retrospective observational study	Adults in South Florida	Geographic/demographic context	BCC and SCC incidence	Reported changing relative incidence patterns of BCC and SCC, contributing contemporary U.S. NMSC epidemiological evidence.
Erickson et al. (2025) [27]	Prospective cohort	52,625 pesticide applicators, AHS	Carbaryl use	Incident cancer	Updated the evaluation of cancer incidence associated with occupational carbaryl use; provides pesticide-specific rather than direct NMSC evidence.
Asgari et al. (2015) [28]	Retrospective cohort	Kaiser Permanente members	Demographic and temporal factors	BCC incidence	Identified temporal trends in BCC incidence and demographic subgroups at increased risk.
Rogers et al. (2015) [29]	Population-based observational study	U.S. population	Population epidemiology	Keratinocyte carcinoma/NMSC incidence	Estimated the substantial incidence of keratinocyte carcinoma in the U.S., establishing the population burden of NMSC.
Cahoon et al. (2015) [30]	Prospective cohort	Nationwide U.S. cohort of women	Estrogen-related and individual factors	BCC incidence	Evaluated individual factors associated with BCC incidence, providing additional context regarding susceptibility to BCC.

Table 3 presents the methodological quality assessment of the included observational studies using NOS [21, 22]. The methodological quality of the included studies was generally moderate to high. Seventeen studies were classified as high quality and nine as moderate quality, with no studies classified as low quality.

**Table 3:** Quality Assessment for observational studies (NOS) Maximum possible score = 9 points: Selection, up to 4 points; Comparability, up to 2 points; and Outcome/Exposure, up to 3 points. Cohort studies were assessed using the cohort version of the NOS, case-control studies using the case-control version, and cross-sectional studies using the adapted NOS for cross-sectional studies [21, 22]. Higher scores indicate better methodological quality. Studies scoring 8–9 were classified as high quality, 6–7 as moderate quality, and ≤5 as low quality. NOS: Newcastle–Ottawa Scale

Study (First Author,Year)	Selection (Max 4)	Comparability (Max 2)	Outcome/Exposure (Max 3)	Total (Max 9)	Quality
Lerro et al., 2015 [1]	4	2	3	9	High
Jones et al., 2015 [2]	4	2	3	9	High
Silver et al., 2015 [3]	4	2	3	9	High
Ransohoff et al., 2016 [4]	4	2	3	9	High
Goldenberg et al., 2016 [5]	4	2	3	9	High
Barton et al., 2016 [6]	4	2	3	9	High
Bonner et al., 2017 [7]	3	1	2	6	Moderate
Muzic et al., 2017 [8]	3	1	2	6	Moderate
Andreotti et al., 2018 [9]	4	2	3	9	High
Nemer et al., 2018 [10]	3	2	2	7	Moderate
Ragan et al., 2019 [11]	3	2	3	8	High
Lerro et al., 2019 [12]	4	2	3	9	High
Lerro et al., 2020 [13]	4	2	3	9	High
Bormann et al., 2020 [14]	3	1	2	6	Moderate
Lukowiak et al., 2020 [15]	3	2	3	8	High
Chiang et al., 2021 [16]	3	2	2	7	Moderate
Shrestha et al., 2022 [17]	4	2	3	9	High
Chang et al., 2023 [18]	3	2	2	7	Moderate
Kao et al., 2023 [23]	3	2	2	7	Moderate
Remigio et al., 2024 [24]	4	2	3	9	High
Tucker et al., 2025 [25]	3	2	2	7	Moderate
Vanaria et al., 2025 [26]	3	2	3	8	High
Erickson et al., 2025 [27]	4	2	3	9	High
Asgari et al., 2015 [28]	4	2	3	9	High
Rogers et al., 2015 [29]	4	2	3	9	High
Cahoon et al., 2015 [30]	4	2	3	9	High

Study findings

The 26 studies included in the evidence synthesis provided different levels of evidence relevant to occupational pesticide exposure and skin cancer risk. Direct evidence was limited, with relatively few studies specifically examining occupational pesticide or agrochemical exposure in relation to skin cancer outcomes. Tucker et al. provided the clearest direct evidence, reporting that dermal exposure to pesticides and fertilizers among U.S. farmers and ranchers was associated with higher odds of physician-diagnosed skin cancer (OR 1.30, 95% CI 1.08-1.56) [25]. This finding supports a possible association between occupational agrochemical exposure and skin cancer, although the observational design does not establish causality or isolate the effects of individual pesticide compounds.

A substantial proportion of the remaining evidence came from the AHS, which has followed licensed pesticide applicators and other agricultural participants over extended periods. These studies examined exposure to specific pesticides, including acetochlor [1], diazinon [2], metolachlor [3], glyphosate [9], dicamba [13], pyrethroids [17], atrazine [24], and carbaryl [27]. Long-term follow-up of the AHS also provided broader information on cancer incidence among pesticide applicators [12]. These investigations are important for understanding the potential carcinogenic consequences of occupational pesticide exposure, but most evaluated overall cancer incidence, site-specific malignancies other than NMSC, or mortality. They were therefore considered supporting evidence rather than direct estimates of pesticide-associated BCC or SCC risk.

The included studies also identified factors that complicate interpretation of the pesticide-skin cancer relationship. UV radiation remains particularly relevant because agricultural workers commonly work outdoors, potentially experiencing substantial solar exposure alongside pesticide exposure [4,11]. Studies of agricultural workers identified variation in sun-protection, skin cancer prevention, and screening practices [11,14]. Population-based investigations also showed that BCC and SCC occurrence varies with demographic and geographic characteristics and individual risk profiles [5,6,8,10,15,16,26,28-30]. These findings emphasize that occupational pesticide exposure occurs within a broader pattern of environmental and individual risk factors.

Evidence concerning biological mechanisms was more limited. Biomonitoring data from the AHS showed associations between glyphosate exposure and urinary biomarkers of oxidative stress [18]. Although this does not demonstrate that glyphosate causes NMSC, it provides biological evidence relevant to mechanisms that may contribute to carcinogenesis. Overall, the evidence suggests that pesticide exposure should not be considered independently of UV exposure, occupational practices, individual susceptibility, and protective behaviors.

Discussion

The findings of this review suggest a possible association between occupational pesticide exposure and skin cancer risk, but the evidence specifically addressing BCC and SCC remains limited. This distinction is central to the interpretation of the review. Tucker et al. directly evaluated dermal agrochemical exposure in relation to skin cancer [24], whereas several other studies examined individual pesticides or occupational pesticide exposure in relation to broader cancer outcomes [1-3,7,9,12,13,24,27]. Other included studies contributed information on NMSC incidence, risk factors, UV exposure, demographic patterns, or occupational prevention practices [4-6,8,10,11,14-16,26,28-30]. These studies strengthen the context in which the direct evidence can be interpreted, but they should not be regarded as independent demonstrations that pesticide exposure increases NMSC risk.

An important challenge is the close relationship between pesticide exposure and other occupational hazards. Agricultural workers who handle pesticides may also spend prolonged periods outdoors and therefore experience substantial UV radiation. This creates difficulty in separating a possible pesticide-associated effect from the established influence of solar exposure. The findings on skin cancer prevention among agricultural workers are particularly relevant because protective practices were not uniform [11,14]. Inadequate or inconsistent protection from sunlight could contribute to observed skin cancer risk independently of pesticide exposure. Differences in age, sex, geographic location, and individual susceptibility may introduce additional variation [5,6,8,10,15,16,26,28-30]. These factors need to be considered when interpreting associations observed in occupational populations.

Differences in pesticide exposure assessment provide another source of uncertainty. The AHS studies generally benefited from large occupational cohorts, prospective follow-up, and detailed pesticide-use information [1-3,9,12,13,24,27]. Nevertheless, the studies differed in the pesticides investigated, duration of follow-up, exposure metrics, and cancer outcomes. Other investigations relied more heavily on participant-reported occupational or behavioral information [14,25]. Such differences reduce direct comparability and make it difficult to determine whether risk varies according to pesticide class, individual active ingredient, duration of exposure, or cumulative exposure.

The biomonitoring evidence provides an additional perspective. The association between glyphosate exposure and oxidative-stress biomarkers reported by Chang et al. [18] offers biological evidence relevant to carcinogenic processes, but mechanistic findings cannot substitute for epidemiological evidence demonstrating an association with BCC or SCC. Future studies would benefit from combining detailed occupational exposure histories with biomonitoring where feasible and from examining BCC and SCC separately. Adjustment for UV radiation, PPE use, occupational characteristics, and other relevant exposures will also be important in determining whether pesticide exposure contributes independently to NMSC risk.

Taken together, the findings do not support a firm causal conclusion. Rather, they identify a possible occupational association that warrants further investigation. The strongest contribution of the current evidence is the combination of a limited direct pesticide-skin cancer signal with a broader body of evidence concerning pesticide exposure, NMSC epidemiology, occupational UV exposure, preventive behavior, and biological plausibility. Prospective studies designed specifically around NMSC outcomes are needed to determine whether particular pesticides, cumulative exposure, or patterns of dermal exposure are associated with BCC or SCC.

Limitations

Several limitations should be considered when interpreting this review. The included studies were heterogeneous in design, pesticide type, exposure assessment, occupational population, duration and frequency of exposure, and outcome definition. Age, sex, UV radiation, PPE use, and other occupational exposures were also inconsistently measured or controlled and may have acted as confounders or effect modifiers. Reliance on self-reported exposure or occupational information in some studies introduced the possibility of recall bias and exposure misclassification. Most importantly, only a subset of the evidence directly examined occupational pesticide or agrochemical exposure in relation to skin cancer, limiting the strength of NMSC-specific conclusions. The substantial clinical and methodological heterogeneity also precluded meaningful quantitative pooling; therefore, a narrative synthesis was undertaken rather than a meta-analysis. Finally, restriction to U.S.-based, English-language studies may limit generalizability and may have introduced language or publication bias.

## Conclusions

This systematic review identified direct and supporting observational evidence relevant to the relationship between occupational pesticide exposure and NMSC risk among U.S. adults. Although a limited subset of studies directly evaluated pesticide or agrochemical exposure in relation to skin cancer outcomes, the broader evidence base provided information on pesticide carcinogenicity, NMSC epidemiology, UV exposure, occupational behaviors, and other potential confounding or modifying factors. Overall, the available evidence suggests a possible association, but substantial heterogeneity and the limited number of direct exposure-outcome studies preclude firm causal conclusions. Additional prospective studies specifically examining pesticide type, cumulative exposure, duration and frequency of application, UV radiation exposure, PPE use, and BCC/SCC-specific outcomes are needed.
